# P2P Lending platforms in Malaysia: the awareness among young adults

**DOI:** 10.12688/f1000research.73401.1

**Published:** 2021-10-28

**Authors:** Lan Thi Phuong Nguyen, Saravanan Muthaiyah, Malick Ousmane Sy

**Affiliations:** 1 Faculty of Management, Multimedia University, Cybejaya, Selangor, 63100, Malaysia; 2 School of Economics, Finance and Marketing, Royla Melbourne Institute of Technology, Melbourne, Victoria, 3000, Australia

**Keywords:** P2P lending platforms, financial literacy, Malaysian young adults, awareness, FinTech, investors

## Abstract

Background - Since 2016, the Securities Commission (SC) in Malaysia has given licenses to only eleven P2P lending platforms. Such lending platforms are expected to disrupt the lending services of traditional lenders in the coming years. However, being still in their infant stages, it is essential to know the extent to which such platforms are made known to potential investors out there. This study examines the extent to which young adults are aware of Malaysia's eleven P2P lending platforms.

Methods - A sample of 65 undergraduate students majoring in finance and accounting was used for this pilot study. An online questionnaire was designed with three main parts: demographic, financial literacy, and P2P lending awareness.

Results - Findings show that more than half of respondents in the sample are not aware of P2P lending platforms in Malaysia.  Most of the respondents are financially literate to certain degrees. Those aware of their presence underestimated the potentially high level of their default rates and misunderstood that investor would be fully protected by such platforms when a loan default.

Conclusions -The study's findings have shed light on the current awareness of P2P lending platforms among Malaysian young adults, potential investors of such platforms in the coming years.

## Introduction

Starting from North America and Europe, P2P lending has grown aggressively in many Asian countries since 2014, taking up its market share even faster than in developed countries ( Stern, Makinen, and Qian, 2017). The availability of the internet and the everyday use of mobile phones in Asia in recent years has open doors for various types of FinTechs platforms, including P2P lending, to come into a country. For instance, China has the most significant number of P2P lending platforms, estimated at 2000 in 2017, according to Stern *et al*. (2017). There are only eleven P2P lending platforms licensed by the Securities Commission (SC) in Malaysia now. Therefore, these platforms need to follow guidelines issued by SC, from which all of them must be incorporated under the Companies Act 1965 with a minimum paid-up capital of RM5 million. In addition, P2P lending platforms cannot place funds received from lenders into their accounts instead of in a third-party account. Furthermore, directors of those P2P lending platforms must prove themselves fit and proper to manage the business. All these requirements help to prevent future fraud caused by these platforms.

To apply for a loan in each P2P lending platform, a borrower needs to apply via its online platform, where information such as payslip, phone number, and other social media profiles are required. Based on such given information, the borrower's risk profile will be analyzed and categorized. Once the borrower's application is accepted, it is open to investors to invest. Unlike the traditional banks, P2P lending platforms do not bear any credit risk of their loans; however, their investors do. Given the standardized set of information (business plan, financial performance, social network status, etc.) required from borrowers, different credit scoring methodologies, different loan disbursement and collection mechanisms at different P2P lending platforms, many questions regarding issues such as security, creditability, and trust of P2P lending platforms, need clear answers to investors, borrowers, and regulators.

Furthermore, the collapse of hundreds of P2P lending platforms in China since 2013 ( Bloomberg Businessweek, October 3, 2018) due to frauds may signal potential risks possessed by P2P lending platforms to investors. In Malaysia, the first P2P lending platform defaulted in August 2018. According to Funding Society Malaysia, this default is mainly because of its SMEs' business slowdown that led to its default payments to the platform (the Edge, September 21, 2018).

Although the default rate for P2P lending platforms remains at 1% and below, as reported by the CEO of Funding Society Malaysia, we do not want to see more P2P lending platforms default in Malaysia soon. Like most Fintech platforms, P2P lending is expected to attract more young adults who are technology savvy and financially literate. This pilot study aims to examine the extent to which the presence of the eleven P2P lending platforms is known to financially literate young adults.

The rest of the paper is organized as follows: Section 2 describes the data sample, study period, and method of analysis used for this study; Section 3 discusses empirical findings and implications of this study; the final section presents main conclusions.

## Methods

Based on the study's objective, an online survey was carried out between January and September 2020. This online survey was approved by the Director of Technology Transfer Office (TTO) and Secretariat of the Research Ethics Committee of Multimedia University. All respondents are MMU university students who were informed with a clear explanation on the questionnaire about the objective of the data collection. Respondents' participation is entirely voluntarily.

Undergraduate students taking finance and accounting courses at Multimedia University, Malaysia, were approached for this pilot study. There are several reasons for this choice of sample selection. First, young adults are more technology savvy and thus are expected to be more aware of the emergence of Fintech, including P2P lending, compared to older people. Secondly, students with finance and accounting knowledge are expected to be more aware of P2P lending than young people with little and no financial knowledge. Thus, results obtained from students with finance and accounting knowledge will provide a somewhat picture of the overall awareness of P2P lending among young people in Malaysia in general. A selected sample of 70 university students majoring in Finance and Accounting answered an online questionnaire consisting of three main parts: demographic, financial literacy, and P2P lending awareness. The first section is the respondents' demographic information, including gender, marital status, age, race, place of residence, and educational background. To measure the financial knowledge of respondents, three big financial literacy questions, created by Global Financial Literacy Excellence Center (GFLEC), are asked in the second section of the questionnaire. These three questions have been used in more than 20 countries around the world to test the level of financial literacy of individuals in three essential areas of knowledge in finance: (1) the effect of compounding interest, (2) the effect of the inflation rate, and (3) the benefit of diversification. The third section of the questionnaire is to collect data on the awareness of the eleven P2P lending platforms. Questions related to the main characteristics of P2P lending given in Shi, Wu, and Hollingsworth (2019) are used to test the awareness of P2P lending platforms in Malaysia. After filtering and validating the collected data, answers from only 65 respondents are used for the analysis. Cross-tabulation analysis and descriptive statistics were employed to analyze the collected data for this pilot study.

### Demographic characteristics of the sample

**Figure 1.**
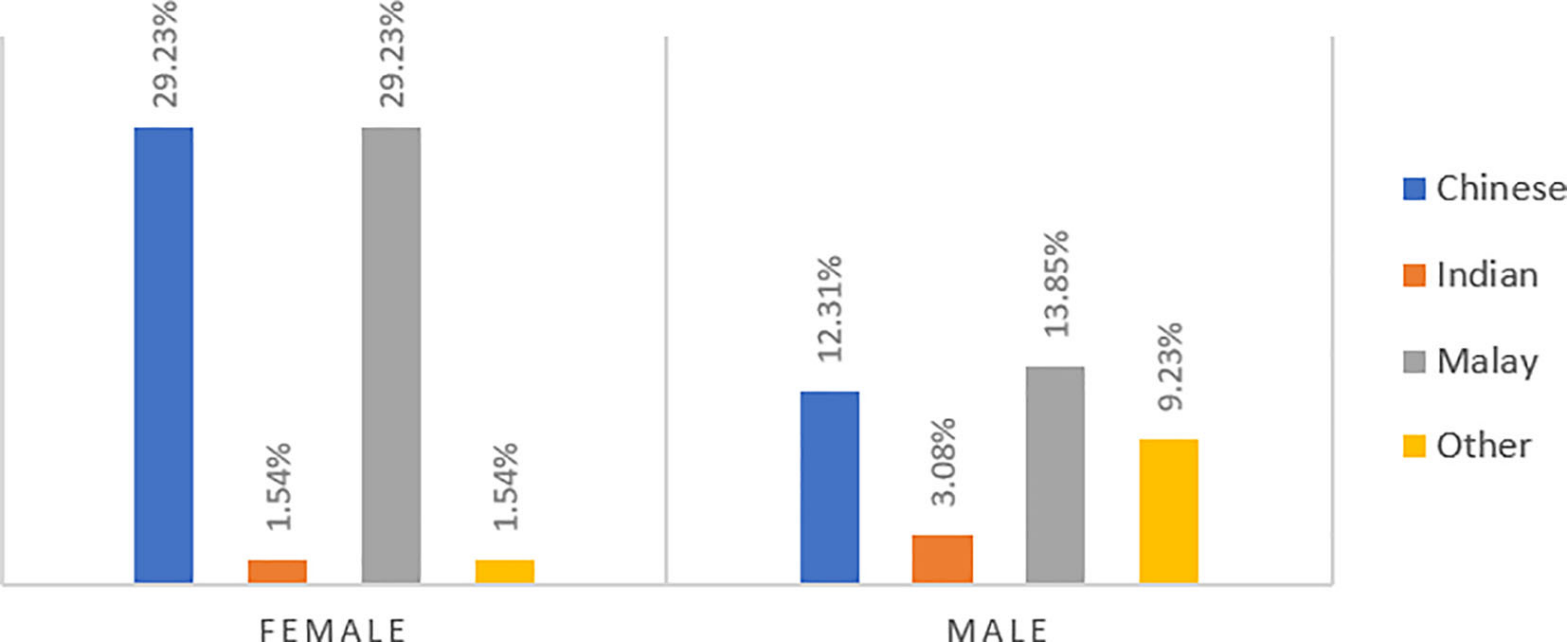
Gender Distribution of respondents in the study sample.

Among the selected 65 undergraduate students, 61.5% are females, and 38.5% are males. In terms of ethnicity, most respondents are Chinese (41%) and Malay (43%), while the remaining are Indian and foreign citizens (see Figure 1). All respondents are from generation Y and are all single. Also, they are all under the age of 50 and are undergraduate students majoring in finance and accounting.

### Assessment of financial literacy

**Figure 2.**
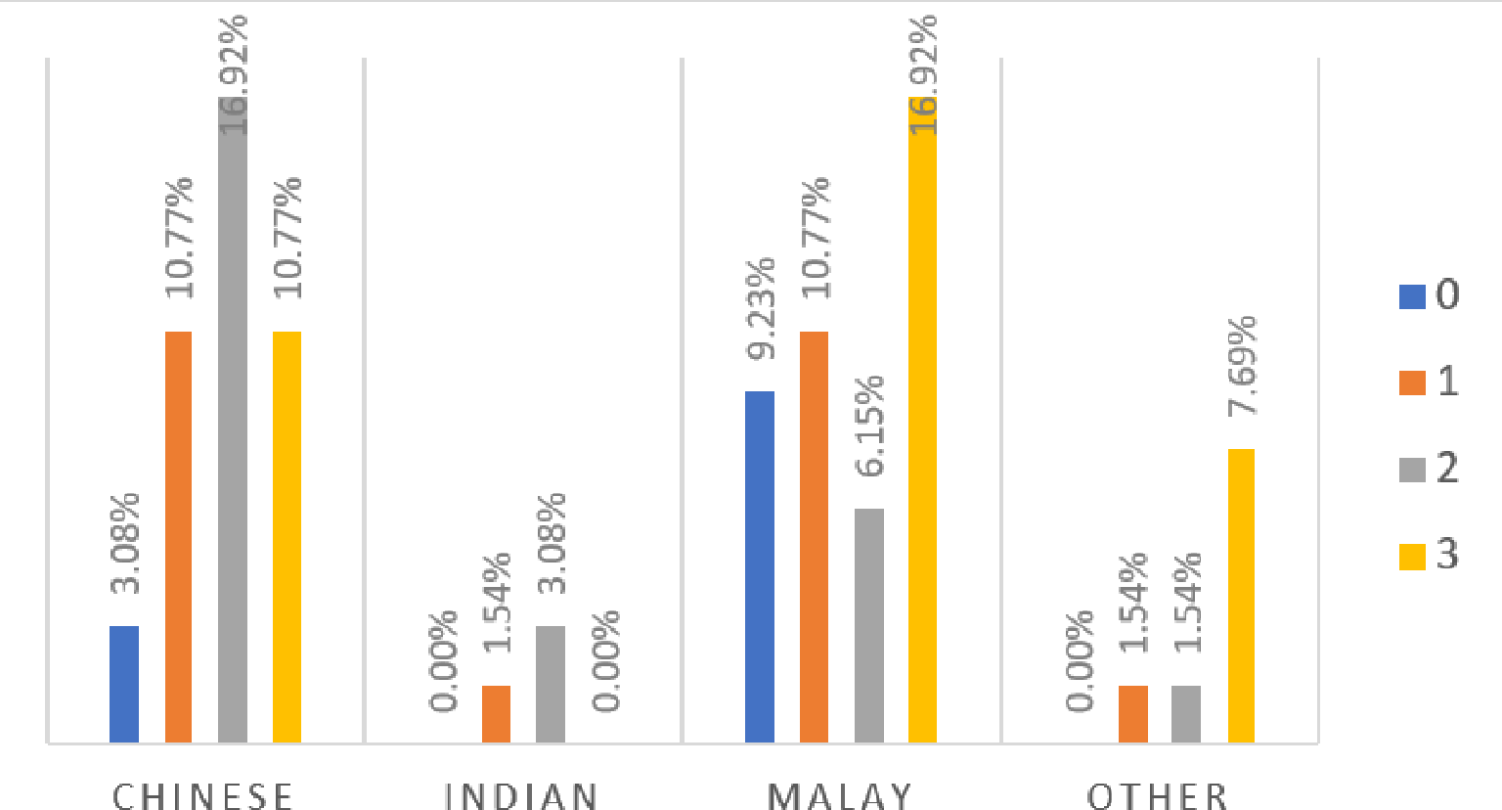
Respondents’ level of financial literacy.

Based on the three answers provided by the respondents, 35.38% of respondents answer all three questions correctly, while 27.69% have two correct answers, and 24.62% have one correct answer. Moreover, 12.31% of them have all three wrong answers (see Figure 2). In short, 87.69% of respondents are all financially literate from low to high degrees. This finding could be because all respondents are undergraduate students taking either finance or accounting degree courses.

### P2P lending platforms awareness

**Figure 3.**
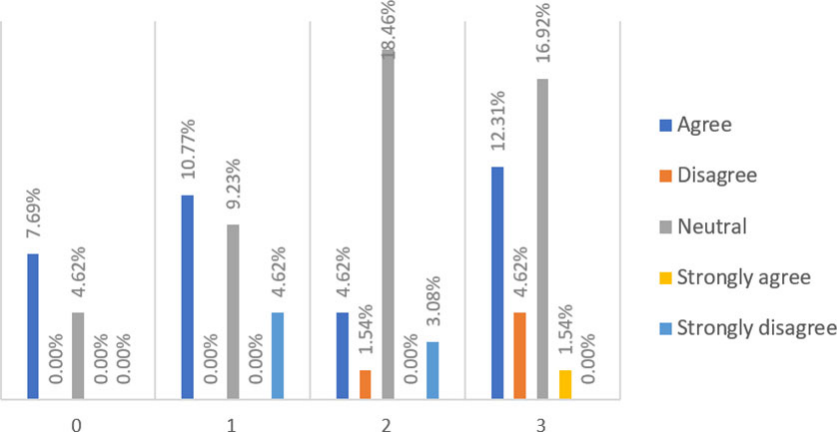
P2P lending is basically a direct lending from lenders to borrowers.

When asked whether P2P lending is a direct lending from lenders to borrowers (see Figure 3), almost half (49.23%) of respondents, among which about 44.62% are financially literate, said they have no idea. However, more (36.92%) people agree and strongly agree with the statement than those (13.84%) who disagree and strongly disagree.

**Figure 4.**
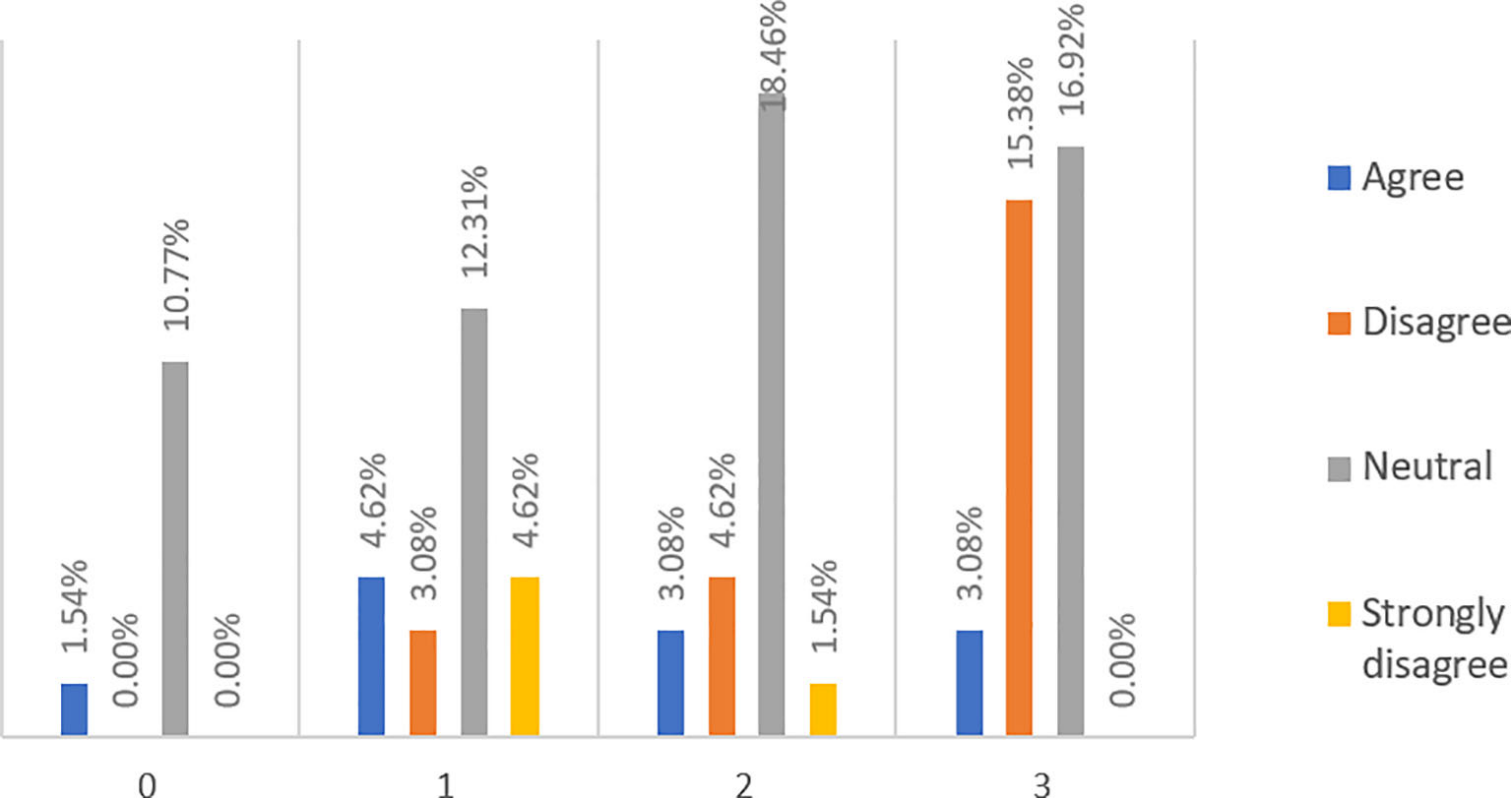
Obtaining loans from banks is much easier than from P2P lending platforms.

When asked if obtaining loans from banks is much easier than from P2P lending platforms (see Figure 4), 58.46% of respondents, among which 47.69% are financially literate, have no idea. However, more respondents (29.23%) disagree and strongly disagree than those (12.31%) who agree with the statement.

**Figure 5.**
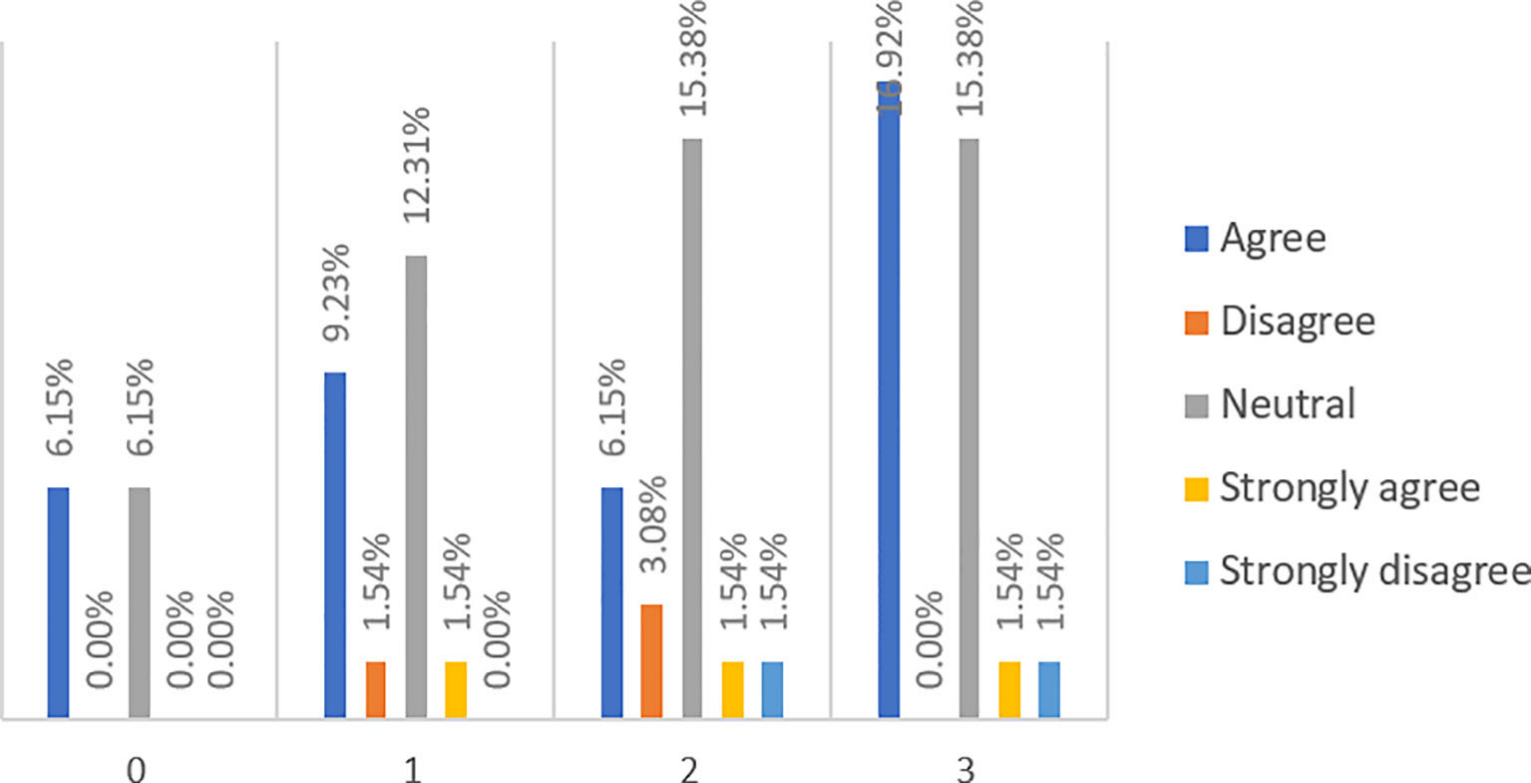
Lending money to banks is safer than to P2P lending platforms.

When asked if lending money to banks is safer than lending money to P2P lending platforms (see Figure 5), 49.23% of respondents, from which 43.08% are financially literate, have no idea. However, more respondents (43.07%) agree and strongly agree than those (7.7%) who disagree and strongly disagree with the statement.

**Figure 6.**
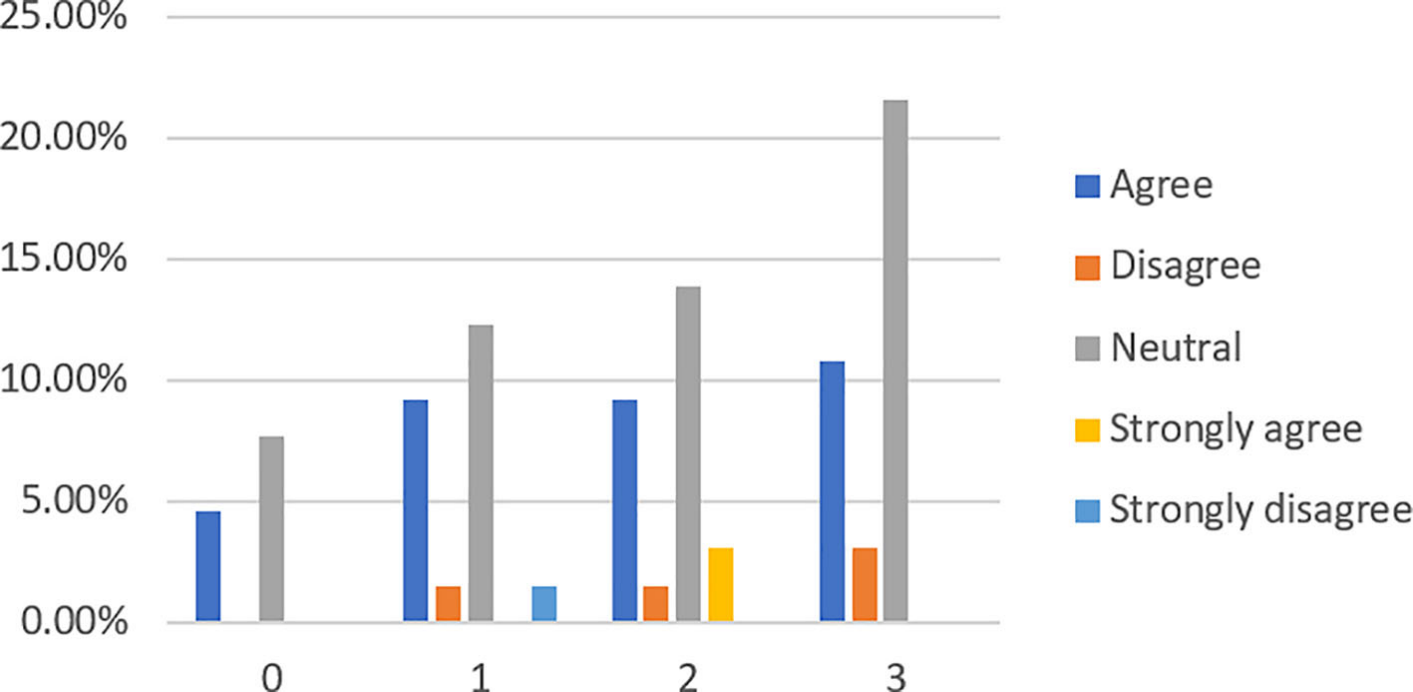
Lenders are protected by P2P lending platforms.

When asked if lenders are protected by P2P lending platforms (see Figure 6), 55.38% of respondents have no idea, from which 47.69% are financially literate. However, fewer respondents (7.69%) disagree and strongly disagree than those (36.92%) who agree and strongly agree with the statement. This finding implies that most respondents are not aware of the straightforward fact that P2P lending platforms do not recover loans for their investors if a loan defaults.

**Figure 7.**
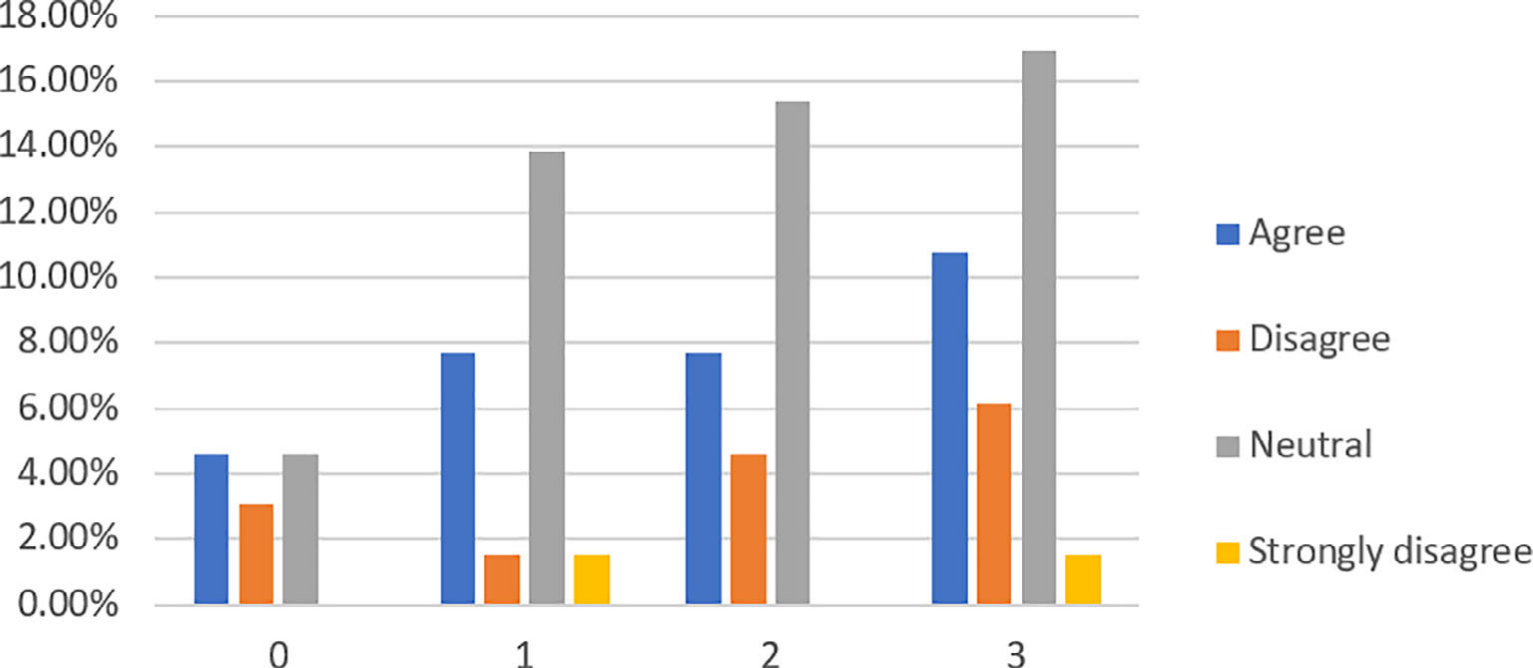
Default risk at P2P lending platforms is perceived to be low and manageable.

When asked if default risk at P2P lending platforms is perceived as low and manageable (see Figure 7), 50.77% of respondents, from which 46.15% are financially literate, have no idea. However, fewer respondents (18.38%) disagree and strongly disagree than those (30.77%) who agree with the statement. Again, this suggests that most respondents underestimate a possible high default rate that can occur at P2P lending platforms.

**Figure 8.**
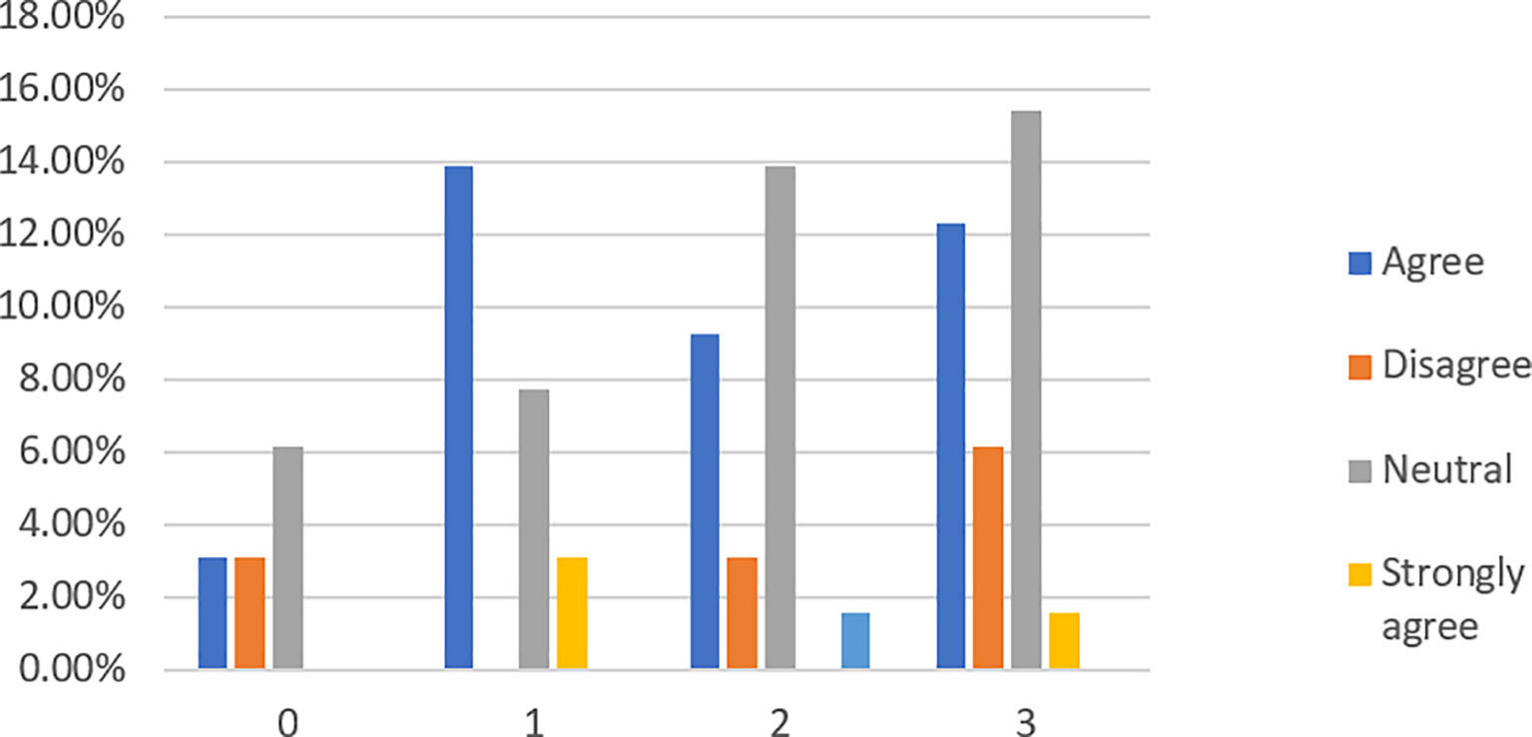
P2P lending platforms offer higher returns to investors than what banks offer.

When asked if P2P lending platforms offer higher returns to investors than what banks offer (see Figure 8), 43.08% of respondents, from which 36.92% are financially literate, have no idea. However, more respondents (43.07%) agree with the statement, while only a smaller number (13.88%) of respondents do not.

**Figure 9.**
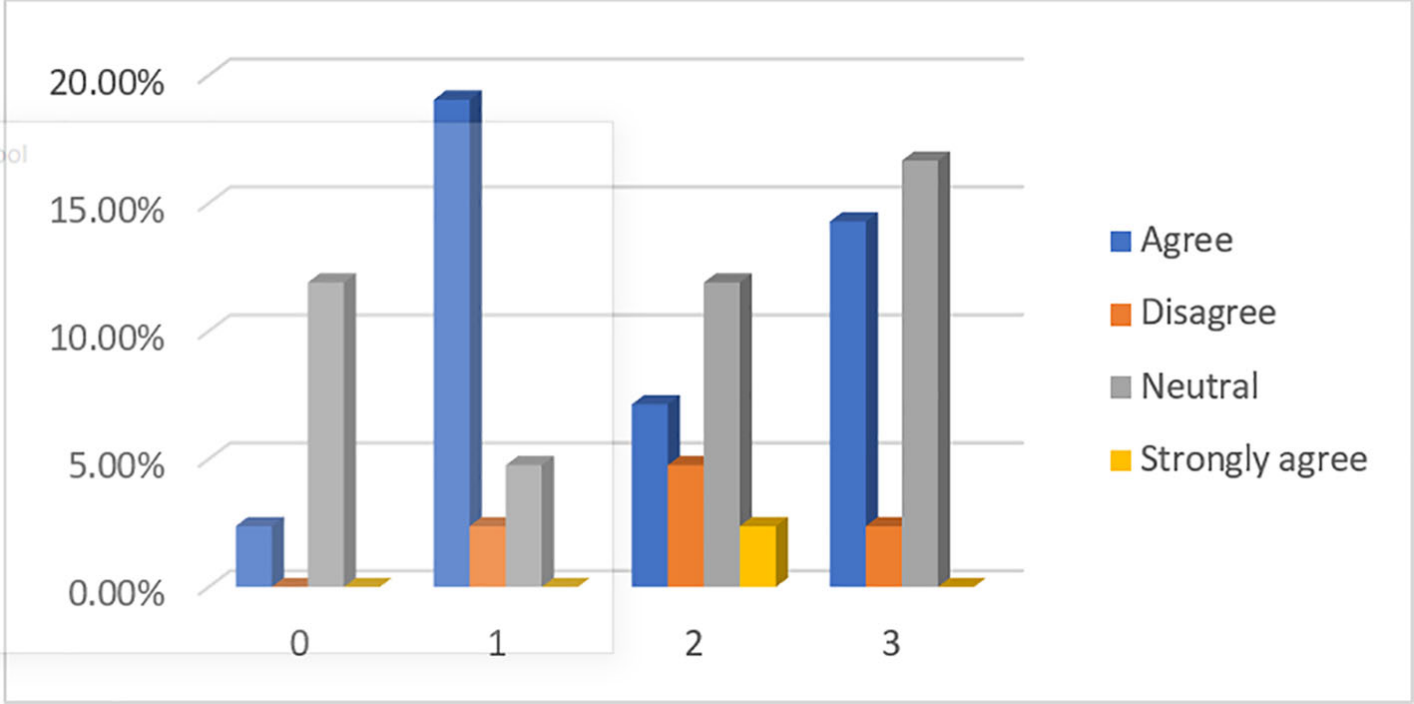
Less documents are needed to get a loan from P2P platforms as compared to banks.

When asked if fewer documents are needed to get a loan from P2P platforms than banks (see Figure 9), 45.24% of respondents have no idea, among which 33.33% are financially literate. However, more respondents (45.24%) think that P2P lending platforms may be much more lenient to borrowers in terms of their loan procedures than those (9.52%) who did not think so.

**Figure 10.**
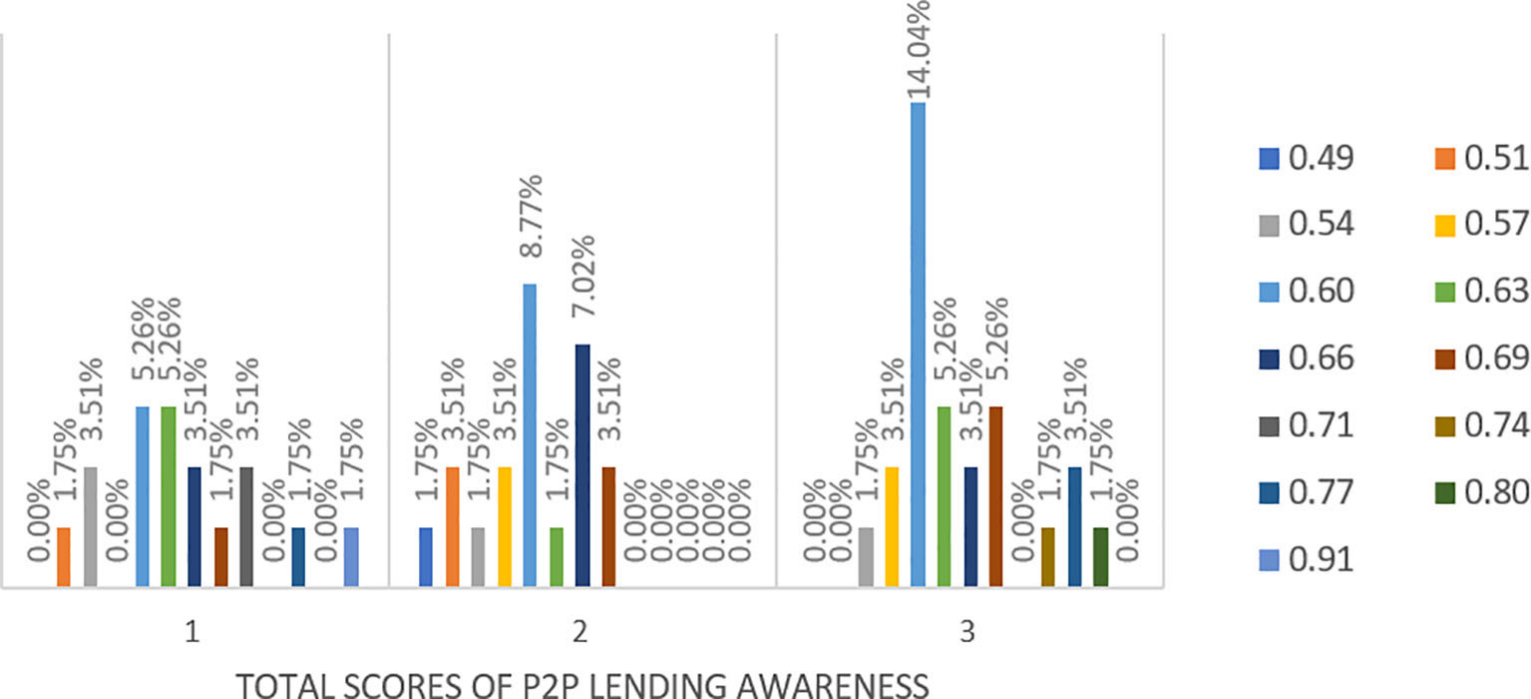
Respondents ‘Overall Awareness of P2P Lending Platforms at Three Level of Financial Literacy.

Based on the seven questions used to test the awareness of P2P lending platforms, scores of awareness are computed, where a 100% score is given to respondents who can give all correct answers for the seven questions. Figure 10 shows only percentages of respondents who have above the average level of awareness, ranging between 60% and 91%, about P2P lending platforms in Malaysia from respondents with low (1), medium (2) and high financial literacy (3). Only 3.5% of respondents have the highest level of awareness, ranging from 80% to 91%. Most respondents (75%), whose scores are from 60% to 77%, are somewhat aware of the presence of P2P lending platforms.

## Conclusion

With the presence of the eleven P2P lending platforms in Malaysia, the lending services of traditional lenders are expected to be disrupted in the coming years. Thus, this study examined the extent to which potential investors in Malaysia know such platforms. Using the non-probability sampling method, a sample of 65 finance and accounting students at Multimedia University, Malaysia, participated in an online questionnaire survey. Most respondents are financially literate at different levels. However, more than half of them had no idea about the presence of P2P lending platforms in Malaysia. Clearly, half of the respondents often could not express their view on the common characteristics of P2P lending platforms. Based on answers related to P2P lending platforms, results show that from 50% to 90% of respondents could answer correctly about some, but not all the main characteristics of P2P lending platforms, i.e., the direct lending function from lenders to borrowers, more straightforward loan assessment, lesser security, lesser document requirement, and higher returns, as compared to banks. However, in terms of loan recovery and default risk at P2P lending platforms, many are not aware of the high potential default rate and the potential zero loan recovery when a loan default occurs at those platforms. As most respondents in the sample are financially literate, lesser awareness about P2P lending platforms in Malaysia may be expected for young adults with lower financial literacy. The overall computed score of financial literacy is relatively low, where only a tiny number of respondents showed their high level of awareness. At the same time, the majority are somewhat aware of the presence of P2P lending platforms. The obtained results shed light on the current awareness of P2P lending platforms in Malaysia among young adults, potential investors of such platforms in the coming years.

## Author contributions

Literature review, research framework, questionnaire design, hypothesis testing and data analysis have been discussed and carried out by all authors of this paper.

## Data availability

Figshare. Data source_P2P Lending Platforms in Malaysia - The Awareness Among Young Adults.xlsx. DOI: https://doi.org/10.6084/m9.figshare.14877381.v1 ( Nguyen, 2021).

This project contains the following data:

- Since 2016, the Securities Commission (SC) in Malaysia has given licenses to only 11 P2P lending platforms. Such lending platforms are expected to disrupt lending services of traditional lenders in the coming years. However, being still in their infant stages, it is important to know the extent to which such platforms are made known to potential investors out there. This study aims to examine the extent to which young investors are aware of the presence of the 11 P2P lending platforms in Malaysia. Using non-probability sampling method, a pilot study was carried out with a sample of 65 undergraduate students, majoring in Finance and Accounting. An online questionnaire was designed with three main parts: demographic, financial literacy, and P2P lending awareness. Findings show that more than half of respondents in the sample are not aware of the existence of P2P lending platforms in Malaysia. Most of respondents are are financially literate at certain degrees. Those who are aware of their presence, underestimated the potential high level of their default rates, and misunderstood that investors would be fully protected by such platforms when a loan is default. Findings of the study shred lights on the current awareness of P2P lending platforms in Malaysia among young adults, who are potential investors of such platforms in the coming years.
