# P2P Lending platforms in Malaysia: the awareness among Malaysian adults

**DOI:** 10.12688/f1000research.73401.3

**Published:** 2023-09-05

**Authors:** Lan Thi Phuong Nguyen, Saravanan Muthaiyah, Malick Ousmane Sy, Wisdom Kalabeke

**Affiliations:** 1 Faculty of Management, Multimedia University, Cybejaya, Selangor, 63100, Malaysia; 2 School of Economics, Finance and Marketing, Royla Melbourne Institute of Technology, Melbourne, Victoria, 3000, Australia; 3 Faculty of Management, Multimedia University, Cyberjaya, Selangor, 63100, Malaysia

**Keywords:** P2P lending platforms, financial literacy, Malaysian young adults, awareness, FinTech, investors

## Abstract

**Background**: Since 2016, the Securities Commission (SC) in Malaysia has given licenses to only eleven P2P lending platforms. Such lending platforms are expected to disrupt the lending services of traditional lenders in the coming years. However, being still in their infant stages, it is essential to know the extent to which such platforms are made known to potential investors out there. This study aims to examine the awareness level of the eleven P2P lending platforms among Malaysian adults. The study also explores if past investment experience and financial knowledge would influence such awareness from Malaysian adults.

**Methods: **A sample of 335 Malaysian individuals was used for this study. An online questionnaire was designed with three main parts: demographic, financial literacy, and P2P lending awareness. Using IBM SPSS Statistics 26, frequency, descriptive, normality, Pearson coefficients and multiple regression analyses were carried out.

**Results**: Although seven out of ten respondents have good knowledge in three areas of finance: compounding rate, inflation and diversification, only 14.33% had a good and excellent awareness level of P2P lending. Thus, one would expect lesser awareness about P2P lending among Malaysian adults whose financial literacy is poor or zero. Test results from multiple regression analysis suggest that past lending experiences positively affect the awareness of P2P lending in Malaysia, but not the financial literacy.

**Conclusions**: The awareness about P2P lending among Malaysian adults is too low, despite their high level of education and financial literacy. No investing experience and not knowing any existing P2P lending in the country may be the reason for this low awareness. Therefore, for P2P lending to thrive in Malaysia, the eleven P2P lending platforms need to be promoted aggressively in various social media outlets.

## Introduction

The availability of the internet and the everyday use of mobile phones in Asia in recent years has opened doors for various types of FinTech platforms, including P2P lending, to come into a country. Starting from North America and Europe, P2P lending has grown aggressively in many Asian countries since 2014, taking up its market share even faster than in developed countries ( Stern, Makinen, & Qian, 2017). For instance, China has the most significant number of P2P lending platforms, estimated at 2000 in 2017, according to Stern *et al*. (2017). There are only eleven P2P lending platforms licensed by the Securities Commission (SC) in Malaysia now. Therefore, these platforms need to follow guidelines issued by SC, from which all of them must be incorporated under the Companies Act 1965 with a minimum paid-up capital of RM5 million. In addition, P2P lending platforms cannot place funds received from lenders into their accounts instead of in a third-party account. Furthermore, directors of those P2P lending platforms must prove themselves fit and proper to manage the business. All these requirements help to prevent future fraud caused by these platforms.

To apply for a loan in each P2P lending platform, a borrower needs to apply for loans via its online platform. Information such as payslip, phone number, and other social media profiles are required on such online platforms. The borrower’s risk profile will be analyzed and categorized based on such given information. Once the borrower’s application is accepted, it is open to investors to invest. Unlike the traditional banks, P2P lending platforms do not bear any credit risk of their loans; however, their investors do. Given the standardized set of information (business plan, financial performance, social network status, etc.) required from borrowers, credit scoring methodologies, loan disbursement and collection mechanisms are different from one P2P lending platform to another. Thus, many questions regarding issues such as security, creditability, and trustworthiness of P2P lending platforms need clear answers from investors, borrowers, and regulators.

Furthermore, the collapse of hundreds of P2P lending platforms in China since 2013 ( Bloomberg Businessweek, October 3, 2018) due to frauds may signal potential risks possessed by P2P lending platforms to investors. In Malaysia, the first P2P lending platform defaulted in August 2018. According to Funding Society Malaysia, this default is mainly because of its SMEs’ business slowdown that led to its default payments to the platform (the Edge, September 21, 2018).

Although the default rate for P2P lending platforms remains at 1% and below, as reported by the CEO of Funding Society Malaysia, we do not want to see more P2P lending platforms default in Malaysia soon. Without a doubt, the successful operation of such P2P lending platforms depends heavily on the awareness of those platforms. This study aims to examine the awareness level of the eleven P2P lending platforms among Malaysian adults. The study also explores if past investment experience and financial knowledge would influence such awareness from Malaysian adults.

The rest of the paper is organized as follows: Section 2 discusses related past studies; Section 3 describes the data sample, study period, and method of analysis used for this study; Section 4 discusses empirical findings and implications of this study; the final section presents main conclusions.

## Literature review

A peer-to-peer lending platform connects borrowers and lenders at a central marketplace for a service fee which is a fraction of the interest paid by borrowers to lenders. With the first two peer-to-peer lending platforms: Zopa and Prosper, launched in 2016, the world of finance has been expanded so that more individuals can participate in lending and borrowing activities. Such a P2P lending platform will carry out its risk classification for each borrower to determine the interest rate charged to that borrower. P2P lending provides many advantages over traditional banks’ lending ( Rubini, 2017). P2P lending allows borrowers to apply for loans online instead of going to a branch; thus, it is convenient and time-saving. Applying for a loan is simple at P2P lending platforms where borrowers only need to express their loan requests, i.e. loan maturity and amount, and provide personal information, i.e. passport/identification card, photo, bank statement. In addition, P2P lending allows more individuals with low or no collateral and lousy past credit records to be financially included. Moreover, individual lenders can allocate even a small amount of money, like RM100 in Malaysia, to individual borrowers and diversify their investments across different borrowers. In short, the P2P lending process is fast and straightforward, where a loan can be granted within a short period as one day.

Although P2P lending provides more opportunities to earn extra income for individual lenders, it exposes them to the potential risk of default. Lenders must be fully responsible for any possible loss when borrowers fail to make payments. Thus, loans provided by P2P lending platforms are categorized as unsecured ( Rubini, 2017). Lenders are advised to make loans to various borrowers instead to mitigate any potential default risk.

In order to make a sound financial decision, an individual is expected to have a certain level of financial literacy, measured by finance concept related knowledge and practical application of such knowledge ( Oanea & Dornean, 2012; Kimiyaghalam & Safari, 2015; Sudakova, 2018; Tavares, Almeida & Cunha, 2019). A person with a higher financial literacy is expected to be more aware of FinTech products, resulting in a higher chance of adopting them ( Morgan & Trinh, 2020; Jin, Seong & Khin, 2018). Thus, basic knowledge about financial concepts is necessary to make a sound lending decision at a P2P lending platform.

A decision making often involves the use of existing knowledge and prior experiences ( Chen, Jin, Zhang & Yang, 2016; Gill, Khurshid, Mahmood & Ali, 2018). Thus, it is believed that past lending experiences that are either good or bad will affect the awareness of new FinTech instruments such as P2P lending.

Undoubtedly, the growth of FinTech is accelerated by the continuous development of technologies, business and personal demands for easy access with lower cost, faster speed and efficiency to various financial transactions ( Shuttlewood, Volin, Wozniak, 2016). According to Gai, Qiu, and Sun (2018), five main issues faced by the FinTech industry, namely security and privacy, are data techniques, hardware and infrastructure, applications and management, and service models. For a layman, personal security and privacy are the two top main concerns that may prevent them from adopting a FinTech instrument like P2P lending ( The Telegraph, 2017). Ultimately, the adoption of FinTech instruments like P2P Lending and others may not be necessarily a smooth ride as it depends heavily on the awareness of individuals living in a community ( Gai, Qiu, & Sun, 2018).

To the best of our knowledge, studies on the awareness of P2P lending platform in Malaysia is somewhat limited to date. Thus, this study aims to fill in this gap.

## Methods

### Survey instrument

Based on the study’s objective, an online survey was carried out between January 2020 and March 2022. This online survey was approved by the Director of Technology Transfer Office (TTO) and Secretariat of the Research Ethics Committee of Multimedia University. Potential respondents for this survey were informed clearly on the questionnaire about the objective of the data collection. Respondents’ participation is entirely voluntarily.

An online questionnaire was designed with four main parts: demographic, financial literacy, and P2P lending awareness. The first section is the respondents’ demographic information, including gender, marital status, age, ethnicity, employment status, and educational background. For gender, female and male respondents are coded as 1 and 2, respectively. In terms of age, 1, 2, 3, 4, 5, and 6 are coded for respondents coming from six age groups, i.e. “17 – 20”, “20 – 29”, “30 – 39”, “40 – 49”, “50 – 59”, and “60 and above”, respectively. In terms of ethnicity, Malay, Chinese, Indian, and other respondents are given a code of 1, 2, 3, and 4, respectively. For educational background, four levels of education, secondary education, diploma, degree and postgraduate/professional, are coded as 1, 2, 3, and 4. For employment status, a code of 1 is given to individuals who are students, while a code of 2 is given to non-student or working adult individuals.

To measure the financial knowledge of respondents, three big financial literacy questions, created by Global Financial Literacy Excellence Center (GFLEC), are asked in the second section of the questionnaire. These three questions have been used in more than 20 countries around the world to test the level of financial literacy of individuals in three essential areas of knowledge in finance: (1) the effect of compounding interest, (2) the effect of the inflation rate, and (3) the benefit of diversification. For each respondent, scores obtained from answers to the three questions will be totalled up and converted to a percentage representing his/her financial literacy score. A correct answer will be given a score of 1, while a wrong answer has a score of 0. The benchmarking scores shown in Table 1 will be used to analyze the level of financial literacy among the respondents.

**Table 1.** Benchmarking scores and levels of financial literacy.

Total financial literacy scores	Total financial literacy percentages	Level
0	0	None
1	33	Weak
2	66	Medium
3	100	Strong

**Figure 1.**
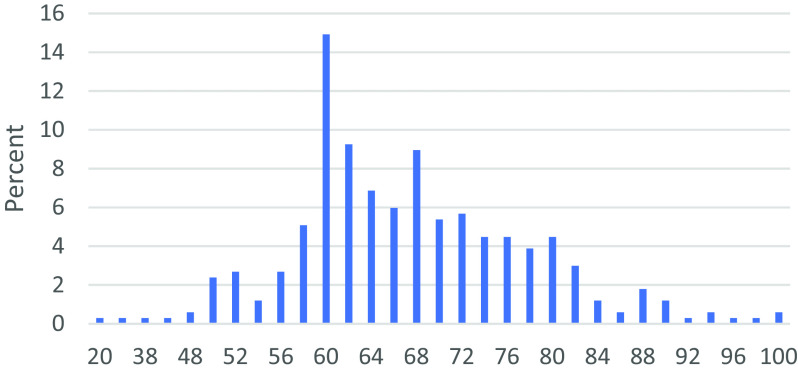
P2P lending awareness scores.

The third section of the questionnaire collects data on the awareness of the eleven P2P lending platforms. Questions related to the main characteristics of P2P lending given in Shi, Wu, and Hollingsworth (2019) and some unique features of P2P lending platforms in Malaysia are used to test respondents’ awareness of P2P lending platforms in Malaysia. Each respondent will be asked if he/she agrees with each of the ten statements describing basic features and characteristics of P2P lending platforms in Malaysia based on a 5-point Likert scale, ranging from strongly disagree to agree strongly. The features and characteristics of P2P lending platforms in Malaysia are (1) the requirements of collateral; (2) the higher interest rates offered as compared to banks; (3) the questionable internet security; (4) the non-standardized credit assessment; (5) the possible default risk, (6) the licensing status; (7) the possible small lending amount; (8) the potentially unrecovered loans, and (9) the presence of unqualified bank borrowers and (10) the free liability of such P2P lending platforms when a loss occurs. The total scores and benchmarking scores for respondents’ P2P lending awareness are computed ( Figure 1) and categorized based on the benchmarking scores given in Table 2.

**Table 2.** Benchmarking scores and levels of P2P lending awareness.

Total P2P lending awareness scores	Total P2P lending awareness percentages	Level
10	20	Very low
20	40	Low
30	60	No idea
40	80	Good
50	100	Very good

### The study sample

Any individuals who have savings and wish to invest for additional income were invited individually to participate in this study using a purposive sampling method. After filtering incomplete questionnaires, the answers of only 335 respondents will be used to analyze this study.

### Statistical analysis

Using IBM SPSS Statistics 26, frequency, descriptive, normality, Pearson coefficients and multiple regression analyses were carried out.

As suggested by Chen, Jin, Zhang and Yang (2016), Gill, Khurshid, Mahmood and Ali (2018), Morgan and Trinh (2020), Jin, Seong and Khin (2020) that prior experience and knowledge and financial literacy are expected to have a positive impact on the awareness of P2P lending. Thus, the multiple regression model will be tested for this study as follows:

P2P Lending Awareness=α+β1Financial Literacy+β2Lending Experience+β2Knowing at least One P2P Lending Platform+εi

Where: α is a constant; β1, β2, and β3 are coefficient correlations for variables of Financial Literacy, Lending Experience and Knowing at least One P2P Lending Platform; and εi is the residual term.

Therefore, the following three hypotheses will be tested:

H _1_: Financial literacy affects the awareness of P2P lending positively.

H _2_: Lending experience affects the awareness of P2P lending positively.

H _3_: Knowing at least one P2P lending platform affects the awareness of P2P lending positively.

## Discussion of findings

### Descriptive statistics and frequency of the study sample

Table 3 and Table 4 reports the frequencies and descriptive statistics of data collected for this study. In terms of gender, 50.7% of respondents are males, and 49.3% are females. The majority of respondents, i.e. 45.4%, are young and between 20 and 29 years of age, while the second (30 – 39 years old) and the third (40 – 49 years old) largest age groups have 24.2% 14.9% of respondents, respectively. In terms of ethnicity, 33.7%, 26.3%, and 14.3% of respondents are Chinese, Malay and Indian, while the rest is specified as others. 79.7% of respondents hold a bachelor’s degree or higher education level. The majority of respondents, i.e. 61%, are single. Out of 335 respondents, 57% are not students, suggesting that they are employable people.

**Table 3.** Frequencies.

		Frequency	Valid percent	Cumulative percent
Lending experience	Yes	136	40.6	40.6
	No	199	59.4	100.0
	Total	335	100.0	
Knowing at least 1 P2P lending platform	Yes	42	12.5	12.5
	No	293	87.5	100.0
	Total	335	100.0	
Financial literacy scores	100	137	40.9	40.9
	67	85	25.4	66.3
	33	72	21.5	87.8
	0	41	12.2	100.0
	Total	335	100.0	
Gender	Male	170	50.7	50.7
	Female	165	49.3	100.0
	Total	335	100.0	
Age	Above 60	8	2.4	2.4
	50-59	25	7.5	9.9
	40-49	50	14.9	24.8
	30-39	81	24.2	49.0
	20-29	152	45.4	94.3
	17-20	19	5.7	100.0
	Total	335	100.0	
Ethnicity	Others	86	25.7	25.7
	Indian	48	14.3	40.0
	Chinese	113	33.7	73.7
	Malay	88	26.3	100.0
	Total	335	100.0	
Education	Postgraduate	84	25.1	25.1
	Degree	183	54.6	79.7
	Diploma	40	11.9	91.6
	Secondary	28	8.4	100.0
	Total	335	100.0	
Maritial status	Married	129	38.5	38.5
	Single	206	61.5	100.0
	Total	335	100.0	
Student/Non-students	Non-student	191	57.0	57.0
	Student	144	43.0	100.0
	Total	335	100.0	

**Table 4.** Descriptive statistics.

	Lending experience	Knowing at least 1 P2P lending platform	P2P lending awareness	Financial literacy	Gender	Age	Ethnicity	Education	Marital	Student/Non-students
N	335	335	335	335	335	335	335	335	335	335
Mean	0.41	0.13	67.27	64.99	1.51	2.80	2.39	2.96	1.39	1.57
Median	0.00	0.00	66.00	67.00	2.00	2.00	2.00	3.00	1.00	2.00
Std. Deviation	0.49	0.33	10.71	35.25	0.50	1.14	1.13	0.84	0.49	0.50
Minimum	0	0	20	0	1	1	1	1	1	1
Maximum	1	1	100	100	2	6	4	4	2	2

When asked if they ever lent money in the past to either friend, family members, businesses, or financial and non-financial institutions, 59.4% said “No,” and 40.6% said “Yes”, suggesting that many respondents have no past lending experience at all. When asked if they could name at least one P2P lending platform in Malaysia, most respondents (87.5%) could not name any.

In terms of financial literacy, 66.3% of respondents have medium (67%) and high (100%) financial literacy, while the rest have weak (33%) or zero (0%) financial literacy. Out of 335 respondents, 137 have a perfect score of 100% for financial literacy in this study, suggesting that these respondents have perfect knowledge in all three areas of finance: compounding rate, inflation and diversification.

Based on the P2P lending awareness scores computed for 335 respondents in the study sample, only 14.33% have a score of 80 and above, suggesting that the majority of respondents in the sample are not aware of the features and characteristics of P2P lending platforms in general. Respondents who could answer correctly for all the 10 questions are classified as having good and very good awareness level accounts for only 14.33% of the study sample.

### Correlation analysis

In terms of Pearson correlation (see Table 5), the P2P lending awareness scores obtained for 335 respondents is significantly and positively correlated with their past lending experience (0.16), ability to name at least one P2P lending platform in Malaysia (0.14), education (0.12), marital status (0.16), and student/non-student status (0.11). This finding may suggest that a person who has one of the following criteria appears to be aware of more features and characteristics of P2P lending:

• previously lent his/her money to others,
• knows at least the presence of one P2P lending platform in Malaysia,
• possess higher education,
• being married, and
• being a working adult

**Table 5.** Pearson correlations between variables.

	Lending experience	Knowing at least 1 P2P lending platform	P2P lending awareness	Financial literacy	Gender	Age	Ethnicity	Education	Marital	Student/Non-Student
Lending experience	1	.128 ^*^	.155 ^**^	-0.012	0.097	0.057	0.088	0.057	0.045	0.018
Knowing at least 1 P2P lending platform	.128 ^*^	1	.135 ^*^	.172 ^**^	-0.006	-0.022	-0.044	.124 ^*^	0.071	0.019
P2P lending awareness	.155 ^**^	.135 ^*^	1	0.066	0.011	0.087	-0.013	.116 ^*^	.157 ^**^	.112 ^*^
Financial literacy	-0.012	.172 ^**^	0.066	1	-0.025	.170 ^**^	-.166 ^**^	.201 ^**^	.166 ^**^	.210 ^**^
Gender	0.097	-0.006	0.011	-0.025	1	0.018	.148 ^**^	-.113 ^*^	-0.042	0.001
Age	0.057	-0.022	0.087	.170 ^**^	0.018	1	-.254 ^**^	.211 ^**^	.609 ^**^	.642 ^**^
Ethnicity	0.088	-0.044	-0.013	-.166 ^**^	.148 ^**^	-.254 ^**^	1	0.034	-.303 ^**^	-.263 ^**^
Education	0.057	.124 ^*^	.116 ^*^	.201 ^**^	-.113 ^*^	.211 ^**^	0.034	1	.173 ^**^	.215 ^**^
Marital	0.045	0.071	.157 ^**^	.166 ^**^	-0.042	.609 ^**^	-.303 ^**^	.173 ^**^	1	.563 ^**^
Student/Non-student	0.018	0.019	.112 ^*^	.210 ^**^	0.001	.642 ^**^	-.263 ^**^	.215 ^**^	.563 ^**^	1

* Correlation is significant at the 0.05 level (2-tailed).

** Correlation is significant at the 0.01 level (2-tailed).

Past lending experience is also positively correlated to the ability to know at least one P2P lending platform in Malaysia at the 5% significance level. This result implies that individuals who were active in lending money in the past will always look for more lending opportunities and thus know about Malaysia’s current P2P lending platform(s).

In addition, the significant Pearson correlations of 0.13 and 0.17 between “knowing at least 1 P2P Lending platform” and each of the “Financial Literacy” and “Education” variables at the 1% and 5% levels, respectively, suggest that individuals with higher education and financial literacy scores also are better at naming at least one P2P platform in Malaysia.

Moreover, the significant and positive correlations between financial literacy and each of the following variables: age (0.17), education (0.2), marital status (0.17), and student/non-student status (0.21) at the 1% level suggest that individuals with older ages or higher levels of education, or being married, or being working adult appear to possess higher financial literacy scores. However, the negative and significant correlation between financial literacy and ethnicity (-0.17) at the 1% level suggests that the highest financial literacy scores were found for Malay respondents, followed by Chinese, Indian and others.

As ethnicity has a significant and negative correlation with each of the following: age (-0.25), marital status (-0.3), and student/non-student status (-0.26) at the 1% level, implying that most Malay respondents are a young student and single, while Chinese and Indian respondents are much older, being married and working. The significant and positive correlation (0.15) between ethnicity and gender at the 1% level hinders that most Malay respondents are female, while more Chinese and Indian respondents are male.

### Multiple regression analysis

The following Table 6 shows the results of multiple regression where the P2P Lending Awareness dependent variable is regressed against three independent variables: Lending Experience, Knowing at Least One P2P Lending Platform, and Financial Literacy. The results show that only past lending experience (0.14) and the knowledge of at least one P2P lending platform (0.1) has a significant and positive impact on the P2P lending awareness at the 1% level. At the same time, there is no significant impact of financial literacy on the awareness of P2P lending. Thus, H1 and H2 cannot be rejected, while H3 is rejected. The findings confirm that an individual with a past lending experience and knowledge of at least one P2P lending platform seems to take the trouble of knowing more about P2P lending characteristics. Thus, past lending experiences and the knowledge of at least one P2P lending platform in Malaysia positively affect the awareness of P2P lending among Malaysian adults. These findings are consistent with what was found in Chen, Jin, Zhang and Yang (2016) and Gill, Khurshid, Mahmood and Ali (2018).

**Table 6.** Multiple regression result.

		Standardized coefficients	t- Statistics	Significance	Collinearity statistics	
		Beta			Tolerance	VIF
Dependent variable: P2P lending awareness	(Constant)		49.512	0.000 ^**^		
	Lending experience	0.142	2.613	0.009 ^**^	0.983	1.018
	Knowing at least 1 P2P lending platform	0.109	1.972	0.049 ^*^	0.954	1.049
	Financial Literacy	0.049	0.901	0.368	0.969	1.032
Model fit results:						
R-square:		0.04				
p-value for F-statistics:		0.004 ^**^				
Mahalanobis distance	Minimum	0.766				
	Maximum	13.969				
Cook's distance	Minimum	0.000				
	Maximum	0.093				

* Significant at the 0.05 level.

** Significant at the 0.01 level.

The insignicant impact of financial literacy on the awareness of P2P lending platform implies that a person with a high financial literacy score may not necessarily be more aware of P2P lending characteristics. In other words, a financially knowledgeable person may not be interested in anything new innovative investment instrument apart from the traditional one, which is contradicting with the findings in Chen, Jin, Zhang and Yang (2016), Gill, Khurshid, Mahmood and Ali (2018), Morgan and Trinh (2020), Jin, Seong and Khin (2020).

The less-than-5 VIF scores for the three independent variables in the model suggest no multicollinearity issue among them. In addition, Mahalanobis Distance with a value (13.96) for a model of three independent variables suggests no significant outlier in the sample. A less than 0.01 p-value for F-statistics suggests that the hypothesis of b1 = b2 = b3 = 0 should be rejected, implying the model fit. A 4% R-square statistic suggests that only 4% of P2P lending awareness of respondents in the sample can be explained by three independent variables, suggesting that many other variables may explain more about the awareness of P2P lending among Malaysian adults.

## Conclusion

Although eleven P2P lending platforms have been licensed by the Securities Commission (SC) in Malaysia since 2016, the awareness level of these platforms is still unknown among Malaysian adults. This study aims to examine the awareness level of P2P lending platforms among Malaysians. With a purposive sampling method, data were obtained from a sample of 335 Malaysian adults between January 2020 and March 2022. Together with descriptive analysis, tested hypotheses of possible impacts on the respondents’ awareness of P2P lending from three factors: (1) financial literacy, (2) lending experience and (3) knowledge of the existence of at least one Malaysian P2P lending platform, were tested. Overall results show that most respondents in the sample are between 20 and 39 years of age (70%) and had at least a bachelor degree (80%). Although almost 60% of respondents were in the workforce, two out of three respondents did not have past lending experience, and four out of five could not name any P2P lending platforms present in Malaysia. Although seven out of ten respondents have good knowledge in three areas of finance: compounding rate, inflation and diversification, only 14.33% had a good and excellent awareness level of P2P lending. Thus, one would expect lesser awareness about P2P lending among Malaysian adults whose financial literacy is poor or zero. Test results from multiple regression analysis suggest that past lending experiences positively affect the awareness of P2P lending in Malaysia, but not the financial literacy. Thus, one may conclude that the awareness about P2P lending among Malaysian adults is too low, despite their high level of education and financial literacy. No investing experience and not knowing any existing P2P lending in the country may be the reason for this low awareness. Therefore, for P2P lending to thrive in Malaysia, the eleven P2P lending platforms need to be promoted aggressively in various social media outlets. The low lending experiences that Malaysians have may hinder the less or absence of lending opportunities given by the traditional banking system. Thus, the growth of FinTech platforms such as P2P lending will allow more individual Malaysians to invest for additional income.

This study mainly focuses on the awareness level of P2P lending among Malaysian adults and its relation to their prior investment experience, knowledge of at least one P2P lending in Malaysia and financial literacy. Therefore, extended research in future can test for other potentially influencing factors such as individual trust, privacy, risk-averse, etc.

## Author contributions

Literature review, research framework, questionnaire design, hypothesis testing and data analysis have been discussed and carried out by all authors of this paper.

## Data availability

Figshare. Data source_P2P Lending Platforms in Malaysia - The Awareness Among Young Adults.xlsx. DOI: https://doi.org/10.6084/m9.figshare.14877381.v1 ( Nguyen, 2021).

This project contains the following data:

• Since 2016, the Securities Commission (SC) in Malaysia has given licenses to only 11 P2P lending platforms. Such lending platforms are expected to disrupt lending services of traditional lenders in the coming years. However, being still in their infant stages, it is important to know the extent to which such platforms are made known to potential investors out there. This study aims to examine the awareness level of the eleven P2P lending platforms among Malaysian adults. The study also explores if past investment experience and financial knowledge would influence such awareness from Malaysian adults. Using a purposive sampling method, a pilot study was carried out with a sample of 355 Malaysian adults. An online questionnaire was designed with three main parts: demographic, financial literacy, and P2P lending awareness.
