# Supplementary material for: P2P Lending platforms in Malaysia: the awareness among Malaysian adults
Source: F1000Res. 2023 Sep 5;10:1096. Originally published 2021 Oct 28. [Version 3] doi: 10.12688/f1000research.73401.3 (PMC9403355; doi:10.12688/f1000research.73401.3)
Supplement: Peer review history

# Peer review: Reviewer response for version 2

**DOI:** 10.5256/f1000research.133920.r139454

**Published:** 2022-08-24

**Authors:** Hassanudin Mohd Thas Thaker

**Affiliations:** 1 Department of Economics and Finance, Sunway University, Selangor, Malaysia

I think the responses are acceptable and reasonable with proper justifications.

Is the work clearly and accurately presented and does it cite the current literature?

Yes

If applicable, is the statistical analysis and its interpretation appropriate?

Partly

Are all the source data underlying the results available to ensure full reproducibility?

Yes

Is the study design appropriate and is the work technically sound?

Partly

Are the conclusions drawn adequately supported by the results?

Yes

Are sufficient details of methods and analysis provided to allow replication by others?

Partly

Reviewer Expertise:

NA

I confirm that I have read this submission and believe that I have an appropriate level of expertise to confirm that it is of an acceptable scientific standard.

# Peer review: Reviewer response for version 2

**DOI:** 10.5256/f1000research.133920.r139455

**Published:** 2022-06-28

**Authors:** Raden Aswin Rahadi

**Affiliations:** 1 School of Business and Management, Institut Teknologi Bandung, Bandung, Indonesia

In overall the study results have been improved, especially by raising the number of respondents significantly to 355. I am satisfied with the results and analysis and conclude that the study can be indexed in its current form.

Is the work clearly and accurately presented and does it cite the current literature?

Yes

If applicable, is the statistical analysis and its interpretation appropriate?

Partly

Are all the source data underlying the results available to ensure full reproducibility?

Partly

Is the study design appropriate and is the work technically sound?

Yes

Are the conclusions drawn adequately supported by the results?

Partly

Are sufficient details of methods and analysis provided to allow replication by others?

Partly

Reviewer Expertise:

Financial Technology; P2P Lending

I confirm that I have read this submission and believe that I have an appropriate level of expertise to confirm that it is of an acceptable scientific standard.

# Peer review: Reviewer response for version 1

**DOI:** 10.5256/f1000research.77049.r124173

**Published:** 2022-04-08

**Authors:** Raden Aswin Rahadi

**Affiliations:** 1 School of Business and Management, Institut Teknologi Bandung, Bandung, Indonesia

As the data involved in this study is quite limited, the results and analysis from the work is also limited and prone to judgment error. I would suggest to enrich the data to reach 100 at least, following the guidelines of Israel (1992) for minimum number of respondents. ^1^ If the authors can get more data, then the result would be much better

**Results **- Findings show that more than half of respondents in the sample are not aware of P2P lending platforms in Malaysia. Most of the respondents are financially literate to certain degrees. Those aware of their presence underestimated the potentially high level of their default rates and misunderstood that investor would be fully protected by such platforms when a loan default. This can happen because of the limited data.

 Only descriptive analysis is presented in this work. A heavy revamping of the paper has to be done, to present the analysis result in a more scientific way.

 Conclusions should be written as follows: RQ answers, implications (theoretical, practical, managerial), implementation plan, recommendations, limitations and future research.

Is the work clearly and accurately presented and does it cite the current literature?

Yes

If applicable, is the statistical analysis and its interpretation appropriate?

Partly

Are all the source data underlying the results available to ensure full reproducibility?

Partly

Is the study design appropriate and is the work technically sound?

Yes

Are the conclusions drawn adequately supported by the results?

Partly

Are sufficient details of methods and analysis provided to allow replication by others?

Partly

Reviewer Expertise:

Financial Technology; P2P Lending

I confirm that I have read this submission and believe that I have an appropriate level of expertise to confirm that it is of an acceptable scientific standard, however I have significant reservations, as outlined above.

## References

[1] : Determining Sample Size. *Florida Cooperative ExtensionService, Institute of Food and Agricultural Sciencesl* .1922; Reference source

# Response

**Published:** 2022-05-11

**Authors:** Thi Phuong Lan Nguyen

**Affiliations:** MMU, Malaysia

The authors' response to the reviewer can be found here:

*https://f1000researchdata.s3.amazonaws.com/linked/421364.Resposes_to_Reviewer%231.docx*

# Peer review: Reviewer response for version 1

**DOI:** 10.5256/f1000research.77049.r98215

**Published:** 2021-11-10

**Authors:** Hassanudin Mohd Thas Thaker

**Affiliations:** 1 Department of Economics and Finance, Sunway University, Selangor, Malaysia

This is an interesting study on P2P lending platforms which explores the topic in an area in which the literature is limited by focusing on Malaysia. However, there are some major issues that need to be resolved.

**Issues**:

 It is unclear what drives the research motivation: what was the goal of the article, what is the state of debate pertaining to this research?

 Literature review: not included. Why is a literature review reported in the paper?

 Methods: how did the authors determine the sample of 65 students? How are they representative of Malaysia’s youngsters? Please provide a justification for why these ones were chosen in particular.

 Furthermore, the authors report information, but I would suggest the authors include analysis. What is your hypothesis about platform awareness? What are the factors that drive awareness of these platforms? Where are your research gaps? What are the current debates pertaining to your topic in the literature?

 Conclusion: at present, it appears to just be a summary of your results. What do the authors think their managerial/theoretical implications are, and what should we take away from those results?

Is the work clearly and accurately presented and does it cite the current literature?

Yes

If applicable, is the statistical analysis and its interpretation appropriate?

Partly

Are all the source data underlying the results available to ensure full reproducibility?

Yes

Is the study design appropriate and is the work technically sound?

Partly

Are the conclusions drawn adequately supported by the results?

Yes

Are sufficient details of methods and analysis provided to allow replication by others?

Partly

Reviewer Expertise:

I did research on crowdfunding and p2p lending as well.

I confirm that I have read this submission and believe that I have an appropriate level of expertise to confirm that it is of an acceptable scientific standard, however I have significant reservations, as outlined above.

# Response

**Published:** 2022-05-11

**Authors:** Thi Phuong Lan Nguyen

**Affiliations:** MMU, Malaysia

The authors' response to the reviewer can be found here:

*https://f1000researchdata.s3.amazonaws.com/linked/421363.Resposes_to_Reviewer%232.docx*
